# TGF-β1 and TGF-β2 abundance in liver diseases of mice and men

**DOI:** 10.18632/oncotarget.6967

**Published:** 2016-01-21

**Authors:** Anne Dropmann, Tatjana Dediulia, Katja Breitkopf-Heinlein, Hanna Korhonen, Michel Janicot, Susanne N. Weber, Maria Thomas, Albrecht Piiper, Esther Bertran, Isabel Fabregat, Kerstin Abshagen, Jochen Hess, Peter Angel, Cédric Coulouarn, Steven Dooley, Nadja M. Meindl-Beinker

**Affiliations:** ^1^ Molecular Hepatology, Department of Medicine II, Medical Faculty Mannheim, University of Heidelberg, Heidelberg, Germany; ^2^ Department of Medicine II, Medical Faculty Mannheim, University of Heidelberg, Heidelberg, Germany; ^3^ Isarna Therapeutics GmbH, Munich, Germany; ^4^ Department of Medicine II, Saarland University Medical Center, Homburg, Germany; ^5^ Dr. Margarete Fischer-Bosch Institute of Clinical Pharmacology, Stuttgart, Germany; ^6^ University of Tuebingen, Tuebingen, Germany; ^7^ Medizinische Klinik 1, Klinikum der Johann Wolfgang Goethe-Universität, Frankfurt am Main, Germany; ^8^ Bellvitge Biomedical Research Institute (IDIBELL) and University of Barcelona. L'Hospitalet, Barcelona, Spain; ^9^ Institute for Experimental Surgery, Rostock University Medical Center, Rostock, Germany; ^10^ Research Group Molecular Mechanisms of Head and Neck Tumors, German Cancer Research Center (DKFZ), Heidelberg, Germany; ^11^ Section Experimental and Translational Head and Neck Oncology, Department of Otolaryngology, Head and Neck Surgery, University Hospital Heidelberg, Heidelberg, Germany; ^12^ Division of Signal Transduction and Growth Control, DKFZ-ZMBH Alliance, German Cancer Research Center (DKFZ), Heidelberg, Germany; ^13^ Institut National de la Santé et de la Recherche Médicale UMR991, University of Rennes, Pontchaillou University Hospital, Rennes, France

**Keywords:** TGF-β isoform, mouse models, HCC, fibrosis, regeneration

## Abstract

TGF-β1 is a major player in chronic liver diseases promoting fibrogenesis and tumorigenesis through various mechanisms. The expression and function of TGF-β2 have not been investigated thoroughly in liver disease to date. In this paper, we provide evidence that TGF-β2 expression correlates with fibrogenesis and liver cancer development.

Using quantitative realtime PCR and ELISA, we show that TGF-β2 mRNA expression and secretion increased in murine HSCs and hepatocytes over time in culture and were found in the human-derived HSC cell line LX-2. TGF-β2 stimulation of the LX-2 cells led to upregulation of the TGF-β receptors 1, 2, and 3, whereas TGF-β1 treatment did not alter or decrease their expression. In liver regeneration and fibrosis upon CCl_4_ challenge, the transient increase of TGF-β2 expression was accompanied by TGF-β1 and collagen expression. In bile duct ligation-induced fibrosis, TGF-β2 upregulation correlated with fibrotic markers and was more prominent than TGF-β1 expression. Accordingly, MDR2-KO mice showed significant TGF-β2 upregulation within 3 to 15 months but minor TGF-β1 expression changes. In 5 of 8 hepatocellular carcinoma (HCC)/hepatoblastoma cell lines, relatively high TGF-β2 expression and secretion were observed, with some cell lines even secreting more TGF-β2 than TGF-β1. TGF-β2 was also upregulated in tumors of TGFα/cMyc and DEN-treated mice. The analysis of publically available microarray data of 13 human HCC collectives revealed considerable upregulation of TGF-β2 as compared to normal liver.

Our study demonstrates upregulation of TGF-β2 in liver disease and suggests TGF-β2 as a promising therapeutic target for tackling fibrosis and HCC.

## INTRODUCTION

Currently, liver transplantation is the only therapeutic option to fight terminal liver failure. The demand for new therapies increases due to the lack of donor organs and enormous economic costs, leading to a major medical problem. In chronic liver disease (CLD), tissue remodeling and wound healing are interrupted, resulting in complex modulation of signaling processes at the cellular and molecular level. Subsequent fibrosis is the onset of hepatic disease development including cirrhosis, HCC, or hepatic failure [1].

TGF-β is a homodimer that exists in three different isoforms (TGF-β1, TGF-β2 and TGF-β3) in mammals. All TGF-β precursor forms are secreted as latent homodimeric complexes, incorporated into the ECM, and activated by proteolytic cleavage [2]. Generally, the β1 and β2 isoforms are closely related and display about 70% amino acid sequence identity [3, 4]. All TGF-β ligands signal through the same receptor signaling systems [5], initiating various downstream signaling pathways [6]. The TGF-β receptors type I, II and III (TGFβR-I, TGFβR-II, and TGFβR-III) are expressed in almost every mammalian cell type, including cancer cells [7]. TGFβR-I and -II are essential for provoking the biological response of TGF-β isoforms [8], but can bind TGF-β3 and TGF-β1 with higher affinity than TGF-β2 [9–11]. TGFβRs-III (Betaglycan and Endoglin) are described to stabilize TGF-βs in a conformation that is optimal for binding to the signaling receptors. Betaglycan binds all TGF-β isoforms with high affinity [9, 10, 12] and significantly enhances the binding efficacy of TGF-β2 to TGFβR-II (reviewed in Massagué [13]). In contrast, Endoglin only binds TGF-β1 and TGF-β3, but not TGF-β2 [14].

The three isoforms have partly overlapping but non-redundant functions and act as potent regulators of cell growth, differentiation and migration. About 60% of mice lacking TGF-β1 already die *in utero* due to deficient endothelial cell differentiation, which underlines its function in embryogenesis [15]. Surviving mice develop severe multi-organ inflammatory responses in the heart, liver, pancreas, and other organs, and show increased numbers of mitochondria in the liver in response to stress [16, 17]. Mainly developmental defects have also been detected in mice lacking TGF-β2, affecting epithelial-mesenchymal interactions, cell growth, extracellular matrix production and tissue remodeling. TGF-β3 knockout mice also exhibit epithelial-mesenchymal interaction perturbances, evidenced in mice by abnormal lung development and cleft palate [18].

TGF-β1 plays a pivotal role in the development of tissue fibrosis where it stimulates the synthesis and accumulation of ECM components and reduces their degradation by matrix metalloproteinases [19]. *In vitro*, TGF-β2 and TGF-β3 have been reported to exert profibrotic effects in fibrosis-related cell types [20, 21] such as fibroblasts. Accordingly, TGF-β2 has been described to be involved in the activation of mesenchymal cells and matrix production in fibrotic livers [22].

In cancer, TGF-β has a bidirectional role: It is involved in the promotion and inhibition of cancer progression mainly depending on the tumor stage [23, 24], with TGF-β1 being the most widely investigated isoform in many human cancers [25]. Less is known about TGF-β2, which was first described to suppress the effects of interleukin dependent T-cell growth [26]. It is released by tumors of several origins including glioblastomas, breast cancer, melanoma, and others [27]. Immunosuppression induced by TGF-β2 is assumed to be a main mechanism by which tumor cells can escape from immune surveillance.

In the liver, all three isoforms are present, but they are not expressed homogeneously in all different cell types [28]. While TGF-β1 expression is often increased in liver cancer, suggesting a tumor-promoting effect [29, 30], little is known about TGF-β2 in this setting. One group has described high stromal expression of TGF-β2 in intrahepatic cholangiocarcinomas (ICC), which was accompanied by poor prognosis [31, 32, 33]. Other preliminary studies including only a few specimen describe all three isoforms as being overexpressed in HCC and proliferating bile ducts of fibrotic livers compared to normal liver [29, 34]. Moreover, overexpression of TGF-β2 and TGF-β3 but not TGF-β1 was found in cholangiocarcinoma [35]. Thus, with research mainly focusing on TGF-β1, to date only a few studies deal with the expression and function of TGF-β2 [36].

In this report, we comparatively analyzed TGF-β2 and TGF-β1 expression and secretion in murine and human hepatic stellate cells (HSCs), hepatocytes and HCC/hepatoblastoma cell lines. We also investigated the dynamics of TGF-β1 and -β2 isoform expression in liver disease progression using several mouse models of different stages of liver disease, as well as expression in human HCC sample cohorts. We demonstrate that both isoforms are expressed in different liver cell types and their expression is elevated during progression of CLD in mouse models. Although TGF-β2 is mostly secreted at lower levels than TGF-β1, its expression patterns largely follow similar profiles. However, the secretion of TGF-β2 exceeded that of TGF-β1 in some HCC cell lines. Our data further indicate a more prominent role of TGF-β2 in biliary-derived liver disease models. Finally, we delineated overexpression of TGF-β2 in human HCC patient cohorts. In conclusion, our data suggests that TGF-β2 probably plays a role in the process of CLD. Targeting TGF-β1 as a therapeutic approach still remains challenging and our findings now provide the encouragement to study TGF-β2 as an alternative promising target for the treatment of liver fibrosis and HCC.

## RESULTS

### Expression of TGF-β1 and TGF-β2 in murine hepatocytes and HSCs

**Figure 1:**
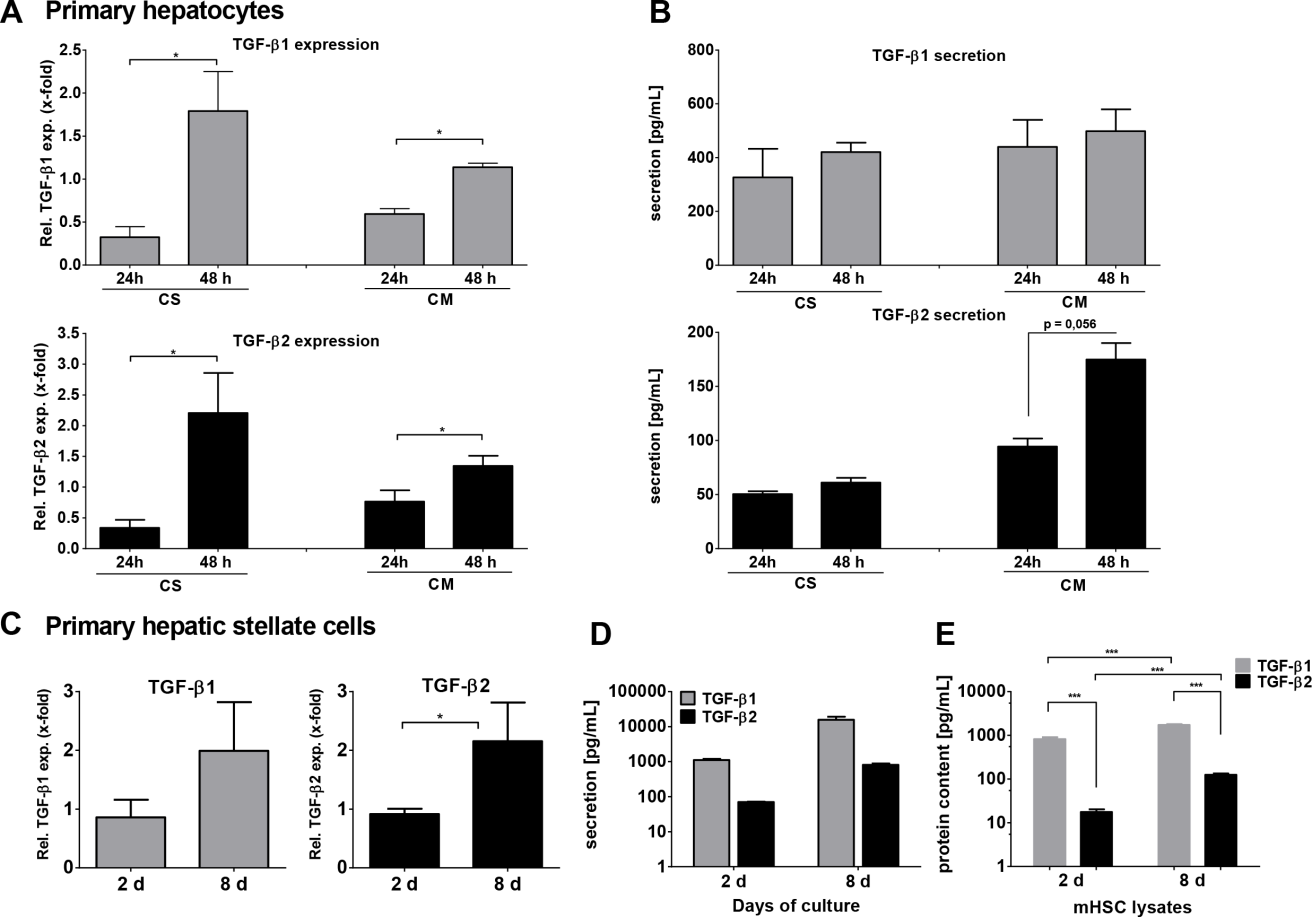
TGF-β1 and -β2 expression and secretion in (A, B) primary mouse hepatocytes after 24 and 48 hours on collagen monolayer (CM) or collagen sandwich (CS) and (C, D) in quiescent (2 days) and culture-activated (8 days) primary mouse HSCs

TGF-β1 and TGF-β2 mRNA expression was first determined in mouse hepatocytes by qPCR (Figure 1A). Hepatocytes were isolated from C57BL/6 wild type mice and cultured either on collagen monolayer (CM) to mimic deteriorating differentiated functions and non-polarized structure, or on collagen sandwich (CS), representing re-established hepatic polarity and stable differentiated functions [37]. In both settings, TGF-β1 and -β2 were increasingly expressed with culture duration after 24 and 48 hours. Induction was higher in CS than in CM for both cytokines. Although secreted at lower levels, the induction of TGF-β2 secretion after 48 h on CM was much stronger than that of TGF-β1 (Figure 1B). The discrepancy between the significant mRNA expression induction of both cytokines and the rather moderate induction of secretion over time suggests storage or intracrine usage of newly synthesized TGF-β in the cells [38] and requires further research.

(**A**) Relative expression of TGF-β1 (upper panel) and TGF-β2 (lower) in hepatocytes was analyzed using qPCR. (**B**) Secretion of TGF-β1 and -β2 by hepatocytes was measured using Quantikine ELISA. Data represent the summary of 3 to 4 independent experiments and error bars represent standard error. After 2 and 8 days, (**C**) TGF-β1 and -β2 expression was determined by qPCR in reference to 2 days cultured HSCs and (**D**) secretion of TGF-β1 and -β2 was measured by Luminex ELISA. (E) Total TGF-β1 and -β2 content was determined in HSC lysates after 2 and 8 days and quantified with Quantikine ELISA. Data are presented as the mean of 3 independent experiments and error bars represent standard deviation.

It is well accepted that upon liver damage HSCs do express and secrete TGF-β1, thus inducing paracrine activation of quiescent HSCs, hepatocyte cytostasis in early stages and tumor-promoting effects in later stages of CLD. Here, we compared TGF-β2 mRNA expression and secretion in quiescent (2 days) and activated (8 days) primary murine HSCs. TGF-β2 mRNA expression, similar to TGF-β1, was increased within 8 days during HSC culture activation (Figure 1C). Furthermore, the secretion of both TGF-β isoforms was demonstrated after 2 days and was increased after 8 days (both ∼10-fold). However, while only about 850 pg/ml TGF-β2 were secreted after 8 days, around 16,000 pg/ml TGF-β1 were detected at that time point (Figure 1D). ELISAs of cell lysates revealed that this divergence corresponded to different levels of protein in the cells. TGF-β1 content was stable after 2 and 8 culture days, but markedly higher than that of TGF-β2 (day 2 ∼200-fold, day 8 ∼10-fold), while intracellular TGF-β2 levels increased about 10-fold between 2 and 8 days (Figure 1E). We conclude from these data the existence of a cytokine specific maximal limit in the cells, which is reached for TGF-β1 already on day 2. Additionally produced cytokines were secreted continuously. Thus, 8-10-fold levels of both cytokines were found in the supernatant as compared to the cell lysates on day 8.

**Figure 2:**
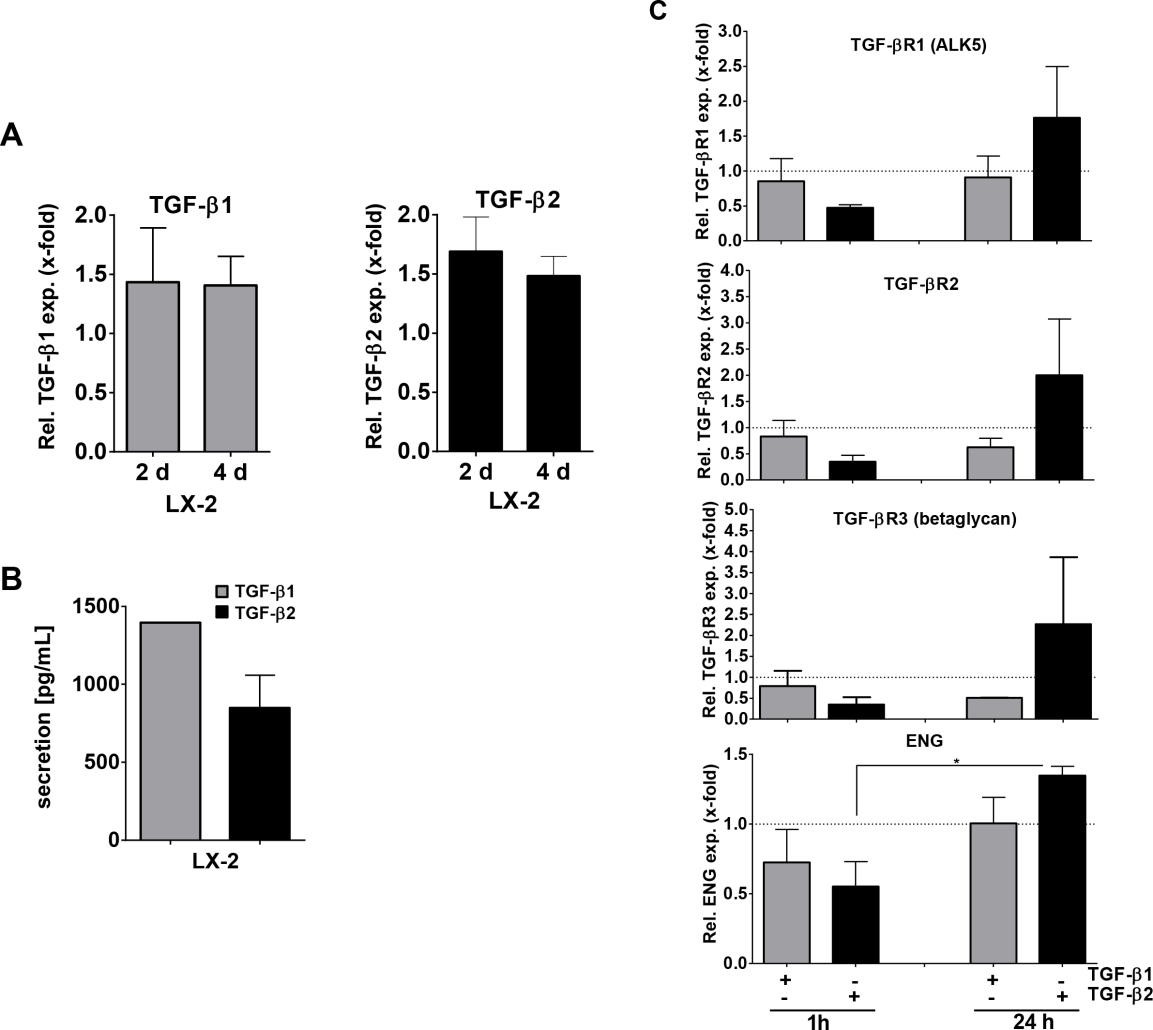
TGF-β1, TGF-β2 and TGF-β-receptor mRNA expression and TGF-β isoform secretion in LX-2 cells (**A**) TGF-β1 and -β2 gene expression after 2 and 4 days and (**B**) secretion after 4 days cell culture. (**C**) qPCR analysis of TGF-β receptor expression after stimulation with 10 ng/ml TGF-β1 or TGF-β2 recombinant protein. Levels of TGF-β receptors in unstimulated cells at the time points indicated served as references.

To translate our results to human HSCs and examine TGF-β1 and -β2 signaling in more detail, we next examined the human HSC cell line LX-2 with respect to TGF-β1 and -β2 expression. We found that both TGF-β1 and -β2 mRNA were expressed in LX-2 cells (Figure 2A). As expected in a cell line, TGF-β mRNA expression was stable when comparing 2 days and 4 days of culture. Secretion of TGF-β1 after 4 days was found to be about 1.5-fold higher than that of TGF-β2 (Figure 2B).

We then analyzed whether TGF-β1 or TGF-β2 secretion impacts on TGF-β signaling in HSCs. As it is known that TGF-β1 can influence the expression of its signaling receptors [39], we analyzed TGF-β receptor expression in LX-2 cells treated with 10 ng/ml TGF-β2 in comparison to TGF-β1. Although LX-2 cells are known to be responsive to TGF-β1, e.g. displaying strong (1α) procollagen upregulation [40], TGF-β1 stimulation of LX-2 cells for 1 h or 24 h either decreased or did not alter TGF-β receptor expression compared to the basal expression levels (Figure 2C, dotted lines). In contrast, TGF-β2 stimulation decreased receptor expression after 1 h, but notably induced TGF-β receptor expression after 24 h (*p*_endoglin_ ≤ 0.027). This suggests that TGF-β2 impacts TGF-β signaling in HSCs in a different manner than TGF-β1. Although Smad1, 2 and 3 phosphorylation efficiency was similar when comparing TGF-β1 and -β2 treatment for 1 or 24 hours, respectively (Supplementary Figure 1A), 24 h TGF-β2 pretreated LX-2 cells were sensitized for subsequent cytokine induced Smad1 signaling (Supplementary Figure 1B), probably due to the receptor upregulation described above. We therefore concluded that besides affecting TGF-β receptor expression, TGF-β2 seems to impact especially pSmad1 signaling. Distinct mechanisms and outcomes (e.g., target gene expression) now need to be delineated.

### Expression of TGF-β isoforms in CCl_4_-induced liver damage

**Figure 3:**
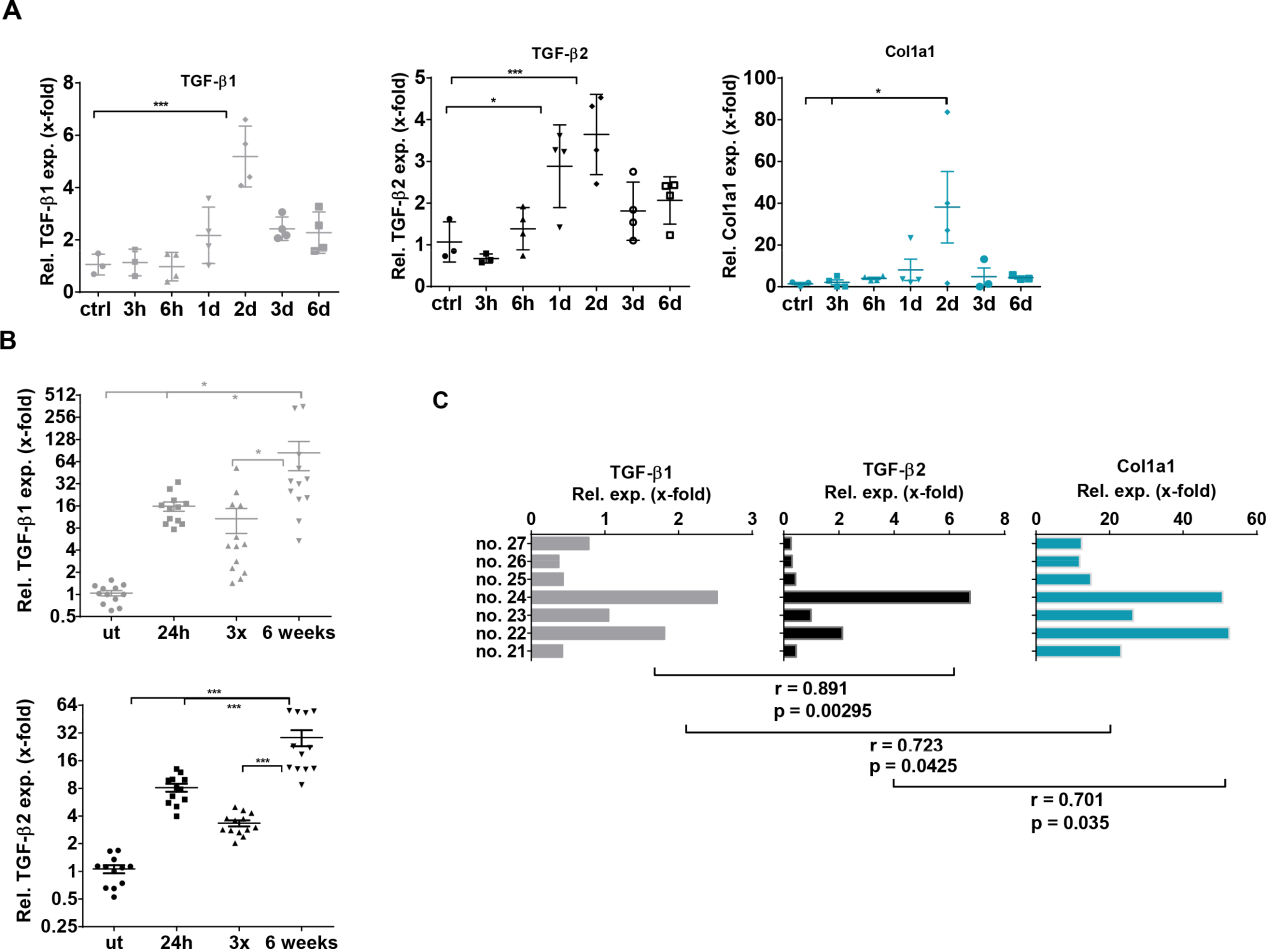
TGF-β1, TGF-β2 and Collagen 1a1 expression in acute and chronic CCl_4_- induced liver damage (**A**) Within six days, expression dynamics of TGF-β1 and −β2 and Collagen 1a1 in the CCl_4_ regeneration model (1 hit) were determined by qPCR in comparison to untreated controls (**B**) TGF-β1 and −β2 mRNA expression was assessed 24 h after 1 CCl_4_ injection, 3 injections within one week or two injections per week for six weeks as indicated. (**C**) TGF-β1 and −β2 expression were correlated with Col 1a1 expression in 7 individual mice with chronic liver damage (6 weeks treatment). Pearson coefficients were r_TGF-β1/TGF-β2_ = 0.891, r_TGF-β1/Col1A_ = 0.723, r_TGF-β2/Col1A_ = 0.701, respectively.

After analyzing TGF-β1 and -β2 expression and secretion in hepatocytes and HSCs, we aimed to translate our findings into *in vivo* models of liver diseases. In a model of liver regeneration upon acute liver damage by CCl_4_, we showed similar dynamics of TGF-β2 and TGF-β1 expression within 6 days by quantitative realtime (q)PCR. Expression of both isoforms peaked on day 2 after CCl_4_ administration. Remarkably, a similarly transient increase in collagen expression was noticed on day 2 (Figure 3A). Encouraged by the correlating behavior of both TGF-β isoforms, we extended this study to chronic liver damage induced by CCl_4_. Mice were treated either with one CCl_4_ injection, with 3 CCl_4_ injections within one week or with chronic treatment twice per week for six weeks. Also in this experiment, CCl_4_ treatment significantly enhanced TGF-β1 and -β2 expression. The highest expression of both isoforms was observed after six weeks of chronic treatment (Figure 3B). Interestingly, expression of both isoforms and collagen positively correlated in individual mice after six weeks of CCl_4_ treatment (Pearson Correlation Analysis *p* < 0.05) (Figure 3C).

### TGF-β isoform expression in models of biliary-derived liver disease

**Figure 4:**
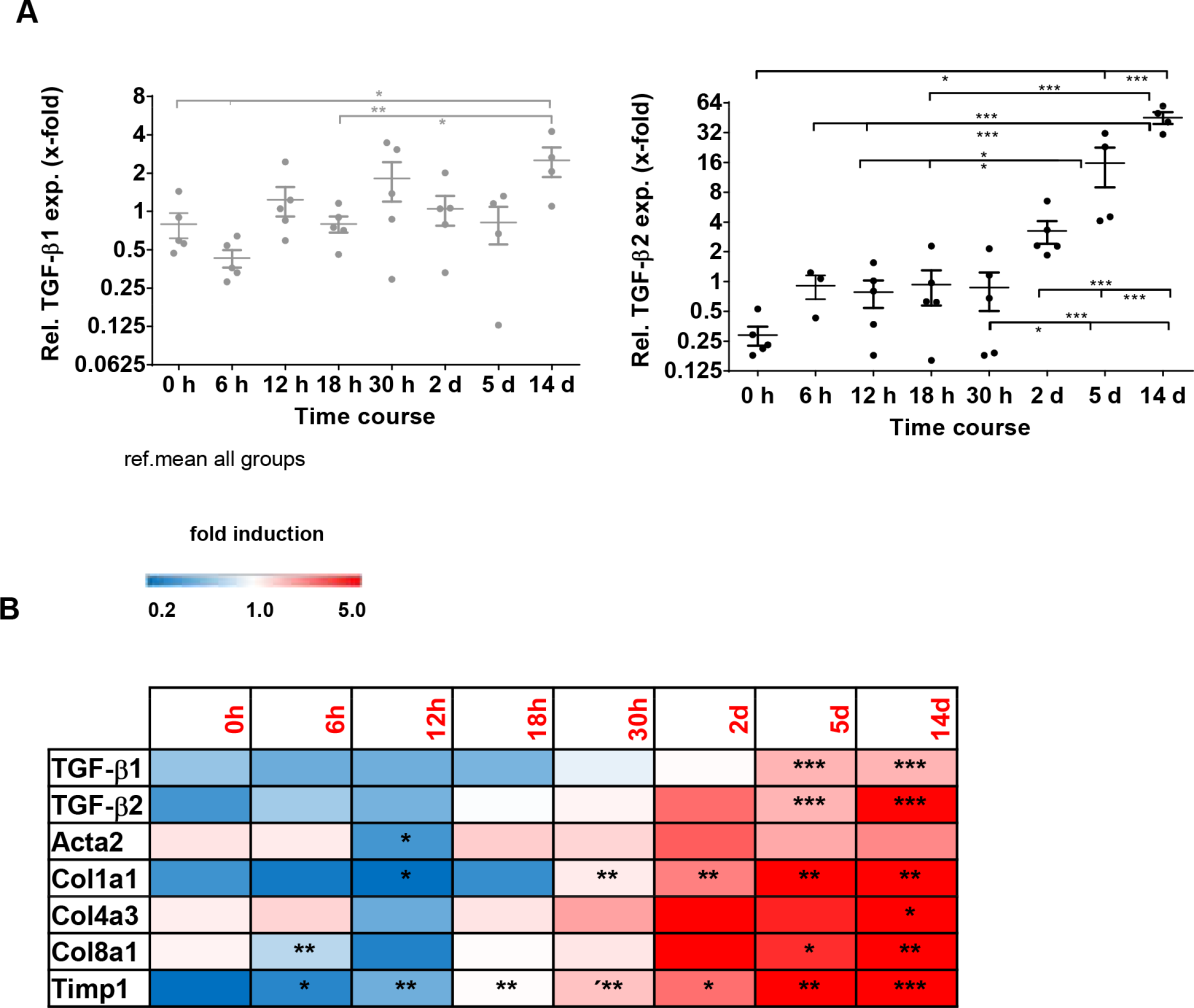
TGF-β1, TGF-β2 and fibrotic marker expression in the bile duct ligation (BDL) model for cholestasis and secondary biliary fibrosis (**A**) TGF-β1 and TGF-β2 expression were determined within a time course of 14 days after bile duct ligation (BDL). Relative expression was normalized to expression of GAPDH and referred to the mean ΔCt of all samples. (**B**) Fluidigm gene expression analysis of typical fibrotic markers displayed in a heatmap. The color represents the expression level of the gene. Red represents high expression, while blue represents low expression. *p < 0.01; **p < 0.005; ***p < 0.001.

To confirm whether the correlating expression pattern of TGF-β1 and -β2 holds true for different etiologies of liver fibrosis, we also analyzed bile duct ligated (BDL) mice as a model for biliary fibrosis. These animals also displayed an elevation of TGF-β1 and -β2 expression within a time course of 14 days after BDL. However, while TGF-β2 was strongly induced after 14 days (∼155-fold as compared to 0 h), there was only a slight induction of TGF-β1 expression during this period (∼4-fold as compared to 0 h) (Figure 4A), indicating a specific and probably more prominent role of TGF-β2 in biliary fibrosis. Time-resolved Fluidigm gene expression analysis revealed parallel induction of TGF-β1, TGF-β2 and different fibrosis markers (Acta2, Col1a1, Col4a3, Col8a1, Timp1) (Figure 4B), underlining the possible involvement of TGF-β2 in the fibrotic process.

**Figure 5:**
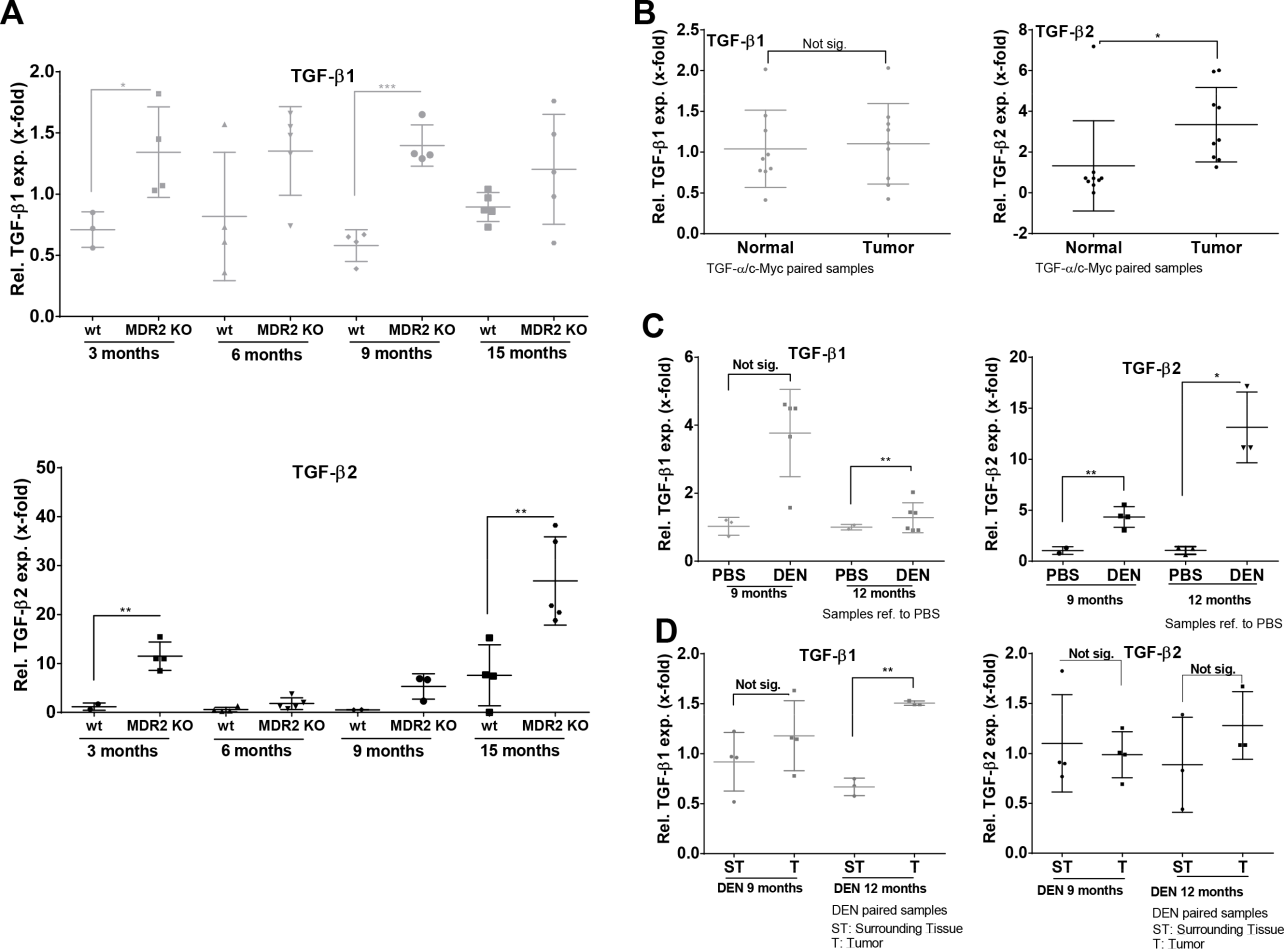
Expression levels of TGF-β1 and TGF-β2 in murine CLD and HCC models (**A**) TGF-β1 (upper panel) and -β2 (lower panel) mRNA expression was examined in MDR2-KO mouse livers after 3, 6, 9, and 15 months and referred to wild type livers of animals at the same age. (**B**) Paired samples of TGFα/cMyc mice were analyzed by qPCR for TGF-β1 and -β2 expression. (**C, D**) In DEN-induced HCC tumor samples from mice, TGF-β1 and −β2 expression was determined and compared to the respective PBS treated controls or surrounding tissue of the same animals.

As TGF-β2 displayed a stronger regulation in the biliary fibrosis model than TGF-β1, we investigated the expression dynamics of both isoforms in MDR2-KO mice, a genetic model for cholestasis-based CLD. Using Fluidigm qPCR, MDR2-KO mice showed consistent TGF-β2 upregulation from 3 to 15 months compared to wild type animals (Figure 5A, lower panel). Although TGF-β1 expression was significantly upregulated at the age of 3 (∼2-fold) and 9 (∼2.5-fold) months in MDR2-KO animals, upregulation of TGF-β1 was notably weaker than that of TGF-β2 (Figure 5A, upper panel). In detail the results were: after 3 months, TGF-β2 ∼10-fold and TGF-β1 ∼1.9-fold; after 6 months, TGF-β2 ∼3-fold and TGF-β1 ∼1.7-fold; after 9 months, TGF-β2 ∼10-fold and TGF-β1 ∼2.4 fold; and after 15 months, TGF-β2 ∼3.5-fold and TGF-β1 ∼1.3-fold.

### Expression of TGF-β1 and TGF-β2 in murine HCC

In MDR2-KO mice, TGF-β2 was strongly upregulated in very early stage CLD (3 months) as well as in late stage CLD (15 months). Generally, it is described that MDR2-KO mice develop cancer after about one year [41]. Thus, we decided to further analyze different HCC mouse models representing late or end stage CLD for TGF-β2 expression. In TGFα/cMyc mice, significantly upregulated TGF-β2 expression but no upregulation of TGF-β1 expression was observed in tumor tissue compared to normal tissue (paired samples) (Figure 5B). TGF-β2 was also upregulated in tumors of DEN-challenged mice compared to untreated controls after 9 and 12 months, but not in comparison to surrounding tissue (Figure 5C–5D). After 12 months, TGF-β1 mRNA was significantly increased in tumors of DEN-treated mice compared to corresponding controls (Figure 5C, left) and in comparison to surrounding tissue of the same animals (Figure 5D, left).

### Expression of TGF-β1 and TGF-β2 in human HCC cell lines

**Figure 6:**
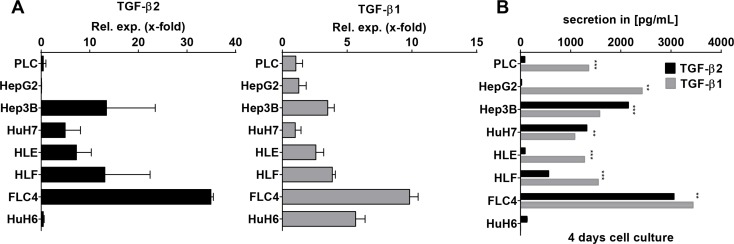
TGF-β1 and TGF-β2 expression pattern in HCC cell lines (**A**) Analysis of TGF-β1 and TGF-β2 expression by qPCR in 7 HCC cell lines and one hepatoblastoma cell line as indicated (**B**) After 4 days in culture, TGF-β isoform secretion was determined performing ELISA. *p ≤ 0.05, **p ≤ 0.01, ***p ≤ 0.001.

As it is most relevant to translate our findings from mouse models to human samples, TGF-β2 and TGF-β1 mRNA expression levels were investigated in 7 different hepatocellular carcinoma cell lines and one hepatoblastoma cell line (HuH6) (Figure 6A). TGF-β2 was expressed in FLC-4, Hep3B, HLF, HLE, and HuH7 cells with decreasing extent in the respective order. HuH6, PLC, and HepG2 expressed very low amounts of TGF-β2. After 4 days of culture, secretion of TGF-β2 was low in PLC, HLE, HepG2, and HuH6 cells, but was high and in a similar range as TGF-β1 in Hep3B, HuH7, HLF, and FLC-4 cells (Figure 6B). Interestingly, in Hep3B and HuH7 cells, TGF-β2 secretion even exceeded TGF-β1 secretion significantly (*p*_Hep3B_ ≤ 6E–05; *p*_HuH7_ ≤ 0.0024). Knockdown of TGF-β2 using a specific antisense oligonucleotide (AON) revealed significant downregulation of the cell number and the expression of the proliferation marker PCNA in HuH7 (Supplementary Figure 2). Whether this accounts for compensatory or tumorigenic proliferations needs to be studied in the future. Together, our data suggest that TGF-β2 plays a noteworthy role in the cytokine signaling of distinct HCC cell lines, which might be independent and have a different outcome than that of TGF-β1.

### TGF-β1 and TGF- β2 expression in cirrhotic and HCC patients

Taken together, the analysis of HCC cell lines revealed significant TGF-β2 expression and secretion of HCC cells and mouse data suggest significant upregulation of TGF-β2 within liver tumor tissue (and not only of TGF-β1). Based on that knowledge, we investigated the occurrence of TGF-β isoforms in human HCC patients. The Oncomine^®^ database was searched in order to analyze TGF-β expression in different HCC patient cohorts. 4 of 7 studies reported TGF-β2 upregulation, whereas only one described TGF-β2 downregulation. TGF-β2 was one of the top 5–10% upregulated genes, while detected downregulation was less prominent (within 25% of downregulated genes). In the same cohorts, TGF-β1 was reported to be upregulated in two studies (within 25% of the most upregulated genes) and downregulated in two other studies (within 25% of the top downregulated genes) (Table 1 and Supplementary Figure 3A). Analyzing cirrhotic patient cohorts via Oncomine^®^, we detected a continuous increase of TGF-β1 in line with disease progression from healthy via cirrhosis to HCC. In contrast, TGF-β2 expression was higher at the cirrhosis stage than in normal liver and even higher than in HCC (Table 2, Supplementary Figure 3B–3C), suggesting a different regulation mechanism of the two isoforms during disease progression (Supplementary Figure 3D).

**Table 1:** HCC sample cohorts used for Oncomine^®^ Research Edition based analysis of TGF-β1 and TGF-β2 expression

TGF-β2	Publication	Samples	Measured Genes Total	Overexpression			Underexpression		
				TGF-β2 Gene Rank	Fold Change (Median Centered Ratio)	*p*-Value	TGF-β2 Gene Rank	Fold Change (Median Centered Ratio)	*p*-Value
1	Chen Liver. Mol Biol Cell. 2002 [76]	197	10.802	5239 (in top 49%)	1.107	0.221	2569 (in top 24%)	−1.258	0.006
2	Guichard Liver. Nat Genet. 2012 [77]	185	18.823	838 (in top 5%)	1.114	1.08E–14	17148 (in top 92%)	1.114	1.000
3	Guichard Liver 2. Nat Genet. 2012 [77]	52	18.823	787 (in top 5%)	1.103	4.32E–6	17195 (in top 92%)	1.103	1.000
4	Mas Liver. Mol Med. 2008 [78]	115	12.603	890 (in top 8%)	1.191	1.56E–6	3547 (in top 29%)	−1.076	0.003
5	Roessler Liver. Cancer Res. 2010 [79]	43	12.603	4250 (in top 34%)	1.194	0.046	5540 (in top 44%)	−1.029	0.118
6	Roessler Liver 2. Cancer Res. 2010 [79]	445	12.624	5015 (in top 40%)	1.053	0.001	5115 (in top 41%)	−1.034	0.004
7	TCGA Liver. No Associated Paper. 2012	212	18.823	792 (in top 5%)	1.233	3.40E–15	ND	ND	ND

TGF-β1	Publication	Samples	Measured Genes Total	TGF-β1 Gene Rank	Fold Change (Median Centered Ratio)	*p*-Value	TGF-β1 Gene Rank	Fold Change (Median Centered Ratio)	*p*-Value
1	Chen Liver. Mol Biol Cell. 2002 [76]	197	10.802	10452 (in top 97%)	−1.557	1.000	512 (in top 5%)	−1.557	1.56E–12
2	Guichard Liver. Nat Genet. 2012 [77]	185	18.823	5712 (in top 31%)	1.013	0.060	12282 (in top 66%)	1.013	0.940
3	Guichard Liver 2. Nat Genet. 2012 [77]	52	18.823	ND	ND	ND	11608 (in top 62%)	1.007	0.820
4	Mas Liver. Mol Med. 2008 [78]	115	12.603	1784 (in top 15%)	1.513	1.40E–4	X	X	X
5	Roessler Liver. Cancer Res. 2010 [79]	43	12.603	1836 (in top 15%)	2.225	3.09E–4	5267 (in top 42%)	−1.071	0.093
6	Roessler Liver 2. Cancer Res. 2010 [79]	445	12.624	5677 (in top 45%)	1.098	0.043	3088 (in top 25%)	−1.111	3.69E–7
7	TCGA Liver. No Associated Paper. 2012	212	18.823	5854 (in top 32%)	1.027	0.021	12130 (in top 65%)	1.027	0.979

Total number of samples, number of genes measured, *p*-values, gene ranks, and the fold change are given for TGF-β1 and TGF-β2 as indicated. Additionally, the table summarizes in which range of differentially expressed genes the respective gene was found in the study (top %). ND = not detected in the study.

**Table 2:** Liver cancer precursor and cirrhosis cohorts used for Oncomine® Research Edition based analysis of TGF-β1 and TGF-β2 expression

No. of cohort in Supplementary Figure 3 B + C	Publication	Reference Tissue
8	Mas Liver, Mol Med. 2008 [78]	Cirhosis vs. normal
9	Wurmbach Liver, Hepatology 2007 [80]	Cirhosis vs. normal
10	Wurmbach Liver, Hepatology 2007 [80]	Dysplasia vs. normal
11	Chiang Liver 2, Cancer Res.2008 [81]	HCC vs. Liver Cancer Type: Liver Cancer Precursor
12	Mas Liver, Mol Med. 2008 [78]	HCC vs. Liver Cancer Type: Liver Cancer Precursor
13	Archer Liver, Cancer Epidemiol Biomarkers Prev. 2009 [82]	HCC vs. Liver Cancer Type: Liver Cancer Precursor
14	Chen Liver. Mol Biol Cell. 2002 [76]	HCC vs. Liver Cancer Type: Liver Cancer Precursor

Number of the respective cohorts in Supplementary Figure 3B and 3C as well as the respective publications and reference tissues are given.

**Figure 7:**
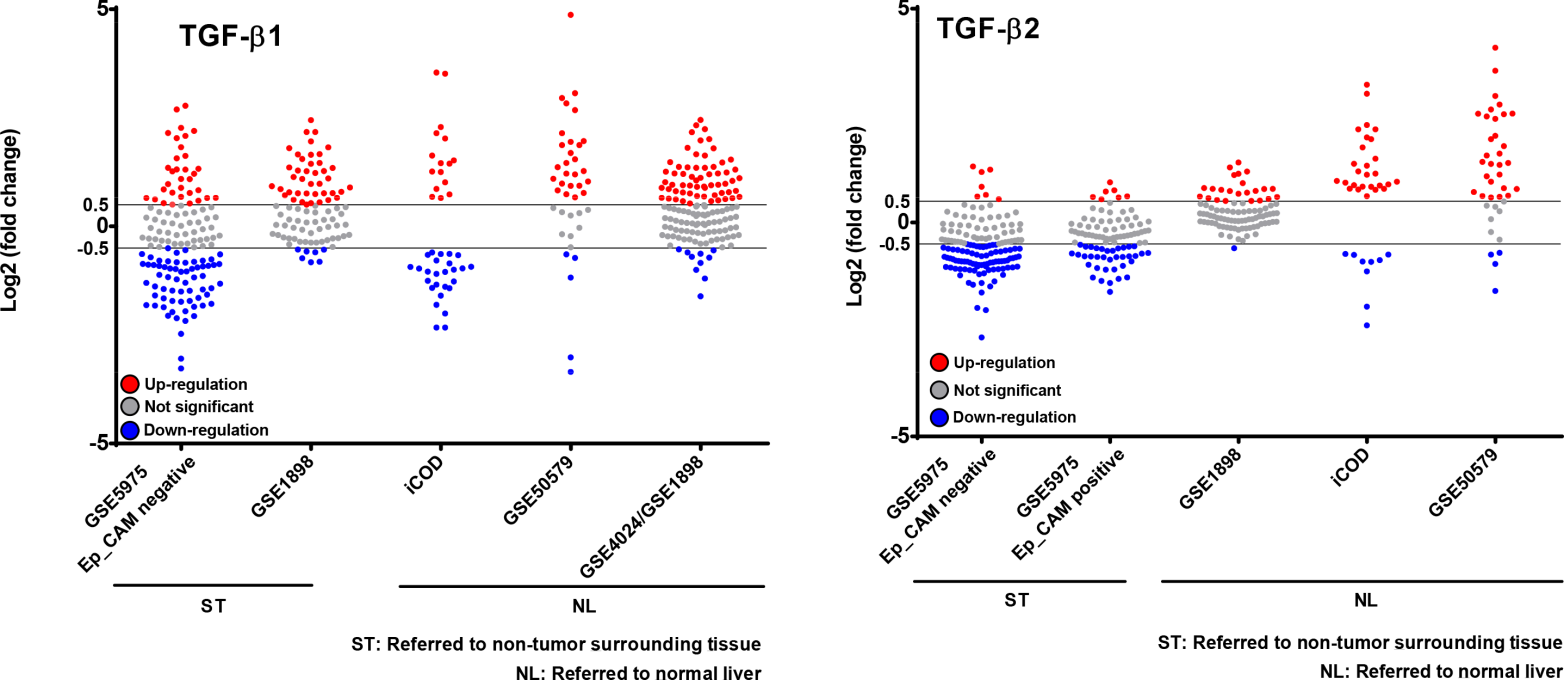
Patient-resolved expression of TGF-β1 and TGF-β2 in different human HCC collectives Free accessible databases (see Table 3) were used to investigate TGF-β1 and TGF-β2 expression in human HCC cohorts. Cohorts with significant *p*-values for TGF-β1 and/or TGF-β2 regulation were taken for further analysis (see Table 4). In individual patients (see single dots) expression changes of TGF-β1 and TGF-β2 were considered to be significant if the fold change (Log2) was ≥ 0.5 or ≤ −0.5.

We then decided to take a closer look at six further HCC cohorts. First, we selected those cohorts that exhibited significant *p*-values (*p* < 0.05) regarding TGF-β1 and/or TGF-β2 regulation in the respective studies (896 patients in total, summaries in Tables 3, 4, 5). It is worth noting that the *p*-value for expression changes provided was neither significant for TGF-β1 in the GSE5975 EpCAM positive and the GSE14520 cohorts, nor for TGF-β2 in the GSE4024/GSE1898 and GSE14520 cohorts. Thus, they were excluded from this analysis. We then applied the selection criteria < −0.5 and > 0.5 Log2 (fold change) to the analysis of individual patients' expression data (single dots). Summarizing the results, TGF-β1 and -β2 were mainly upregulated compared to normal liver; however, a downregulation of TGF-β2 was obvious compared to surrounding tissue of the tumors (Figure 7). This implies that tumor-surrounding tissue itself is significantly altered as compared to normal liver and already displays changed TGF-β signaling signatures. Due to these and other specific changes of the tumor environment, which are known to impact tumorigenesis, expression changes need to be carefully investigated dependent on the context. Interestingly, analyzing patients with defined TGF-β2 regulation in GSE1898/4024, we found a correlation between high TGF-β2 expression and a poorer survival rate (*p* < 0.01) (Supplementary Figure 4), but not with other clinicopathological parameters including AFP levels, tumor size, differentiation grade, cirrhosis, and hepatoblast vs. hepatocyte subtype (data not shown), further suggesting tumor-promoting effects of TGF-β2.

**Table 3:** HCC sample cohorts used for analysis of TGF-β1 and TGF-β2 expression

Publication	Array Name	N°HCC Patients	HCC Etiologies	Normal Liver	Surrounding Tissue
Roessler S. Cancer Res. 2010 [79]	GSE14520	247	HBV		247
Wang XW. Clin Cancer Res., 2007 [83]	GSE5975	236	Ep_CAM +/−; HBV+; Cirrhosis +/−		236
Thorgeirsson S. Nature Genetics 2004 [84]	GSE1898	91	HBV+; HCV+; co-infection; HBV/Alcohol; HCV/Alcohol, Hemochromatosis		91
Neumann O. Hepatology 2012 [85]	GSE50579	40	HBV+; HCV+; co-infection; Alcohol; Cryptogenic; Hemochromatosis; Others	7	
Shimokawa K. BMC Genomics. 2010 [86]	iCOD	140	HBV+; HCV+; co-infection; Alcohol; HCV+ Alcohol, Diabetes; Cirrhosis; Unknown	NA	
Thorgeirsson S. Nature Genetics 2004 [84] Lee JS et al. Nat Med 2006 [87]	GSE4024/GSE1898	142	HBV+; HCV+; co-infection; Alcohol; Cryptogenic; Hemochromatosis; ALD; NASH; Adenoma; Autoimmune; Unknown; Others	10	

NA = not announced in the study.

**Table 4:** Significance of TGF-β1 and TGF-β2 expression changes in different HCC sample cohorts

TGF-β1	N°HCC Patients	adj. *p*-value	Mean Log2 (Fold Change)
GSE1898	91	2,53651E–07	0,47
GSE5975 EpCAM negative	142	0,00090135	−0,34
GSE5975 EpCAM positive	94	(0,36864697)	−0,08
iCOD	140	NA	−0,72
GSE50579	40	0,008	1,13
GSE4024/GSE1898	142	0,05	0,67
GSE14520	247	(0,35)	0,04

TGF-β2	N°HCC Patients	adj. *p*-value	Mean Log2 (Fold Change)
GSE1898	91	1,01742E–07	0,25
GSE5975 EpCAM negative	142	6,0118E–21	−0,57
GSE5975 EpCAM positive	94	2,25993E-09	−0,36
iCOD	140	NA	0,74
GSE50579	40	0,0001	1,42
GSE4024/GSE1898	142	(0,83)	−0,03
GSE14520	247	(0,36)	0,01

Number of patients, *p*-values and fold change of TGF-β1 and TGF-β2 expression in the respective patient cohorts are listed. Only those cohorts with significant *p*-values were used for further analysis (See Figure 7). Non-significant *p*-values are given in brackets. NA = Not announced.

**Table 5:** Detailed analysis of HCC patient cohorts given in table 3 in regard to TGF-β1 and TGF-β2 up- and down-regulation

TGF-β1	N°patients Up-Reg.	N°patients Down-Reg.	% patients Up	% patients Down	%patients not Significant	Mean Log2, Fold Change Up	Mean Log2, Fold Change Down
GSE1898	41	7	45,05	7,69	47,25	1,16	−0,66
GSE5975 EpCAM negative	65	33	45,77	23,24	30,99	1,26	−1,34
iCOD	16	26	11,43	18,57	70	1,45	−1,04
GSE50579	26	5	65	12,5	22,5	1,69	−1,78
GSE4024/GSE1898	61	10	42,96	7,04	50	1,02	−0,71

TGF-β2	N°patients Up-Reg.	N°patients Down-Reg.	% patients Up	% patients Down	%patients not Significant	Mean Log2, Fold Change Up	Mean Log2, Fold Change Down
GSE1898	23	1	25,27	1,1	73,63	0,79	−0,60
GSE5975 EpCAM negative	7	83	4,93	58,45	63,38	0,91	−0,95
GSE5975 EpCAM positive	7	36	7,45	38,3	54,26	0,68	−0,86
iCOD	27	9	19,29	6,43	74,29	1,38	−1,16
GSE50579	29	4	72,5	10	17,5	1,76	−1,01

Only those cohorts with significant *p*-values for the respective genes are used for this analysis, thus excluding TGF-β1 regulation in the GSE5975 EpCAM positive and GSE 14520 cohort as well as TGF-β2 in the GSE4024/GSE1898 and GSE 14520 cohort (see Table 4).

## DISCUSSION

In liver disease, TGF-β1 is described as a key player in the activation of myofibroblasts [42], hepatocyte apoptosis, and proliferation control [43]. Over the decades, its involvement in different stages of CLD and cancer has been investigated and discussed on a broad basis, and a lot of information has been gathered since then. However, the TGF-β family consists of three isoforms with overlapping, but not redundant functions[24].

We comparatively investigated TGF-β1 and -β2 expression in liver cells and diseased livers in various stages of progression. Probably due to more sensitive detection methods, we found that just like TGF-β1, TGF-β2 is expressed in and secreted by both, healthy and diseased hepatocytes and HSCs. This is in contrast to some basic results reported about 25 years ago, where weak TGF-β1 expression was only found near central veins and TGF-β2 was only expressed in bile duct epithelial cells [44, 45]. However, those studies performed in the 1990s focused on the differences in isoform expression between different liver cell types. This led to underestimation of low expression or low signals were not interpreted as significant in comparison to strong signals, e.g., in Kupffer and bile duct cells.

In various models of regeneration, acute and chronic liver disease presented here, we were able to confirm knowledge of the expression patterns of TGF-β1 in liver disease. Reported information available on TGF-β2 in CLD and HCC is less comprehensive so far; however, it implies that TGF-β2 also acts in a profibrogenic and tumorigenic manner in different organs, including the liver. Wang et al.[46] demonstrated that i.p. administration of TGF-β2 in conjunction with CTGF - but not by itself - induced fibrosis of almost all abdominal organs, including the liver. This effect was rather systemically than organ-specific, but multifocal fibrosis of the hepatic capsule plus multiple foci of mild parenchymal fibrosis were detected. Since then, the role of TGF-β2 in fibrogenic mechanisms could be related to a variety of signaling mechanisms, which are partly based on canonical Smad2/3 signaling. In patients with chronic Hepatitis C and advanced fibrosis, upregulation of TGF-β2 correlated well with Smad2 expression [47]. Another indication of canonical TGF-β2 signaling was provided by Wang et al. [48], when they observed diminished TGF-β2 expression and subsequent Smad3 activity upon miR-200a treatment in renal fibrogenesis.

However, non-canonical signaling and crosstalk of TGF-β2 have also been reported by different groups. A Wnt-TGF-β2 axis was discovered in muscle stem cells [49] and a study performed by Sun et al. [50] revealed TGF-β2 and β-Catenin as the two main functional targets of miR-200a expression in hepatic fibrosis. Further, Dong et al. uncovered a universal organ size control mechanism in metazoan, showing that Yap overexpression in the liver increased liver mass in transgenic mice extensively and strongly induced TGF-β2 expression [51, 52]. Together, these findings indicate possible mechanistic links for TGF-β2 and provide rationales for investigating these pathways in liver disease of different origins.

Although our data do not provide experimental evidence for mechanistic details so far, we have shown that in liver regeneration processes in which proliferation and apoptosis events need to be tightly controlled, TGF-β2 is upregulated. During regeneration, expression of both isoforms of TGF-β peaked after day 2, coincident with collagen expression. For TGF-β1, this has been known for a long time; however, our data imply a similar importance of TGF-β2 in these regenerative processes. After repeated chemical intoxication, both TGF-β isoforms were further elevated simultaneously, suggesting a parallel and maybe synergistic function of the two isoforms in CCl_4_-induced progression of liver damage.

In contrast to this concordant behavior in CCl_4_ intoxication related liver disease, TGF-β2 expression patterns differed from those of TGF-β1 in biliary-derived models of liver damage. Interestingly, TGF-β2 was more significantly elevated in mice with bile duct ligation and at different time points of liver disease progression in MDR2-KO mice. This suggests a specific role of TGF-β2 in the development of biliary-derived liver damage, which was already implicated in early results obtained by Milani et al., who described more prominent expression of TGF-β2 than TGF-β1 in bile duct epithelial cells, and particularly high TGF-β2 levels in proliferating bile ducts of fibrotic livers [53]. While a small study including eight twins (E-MTAB-2347) did not reveal TGF-β2 deregulation in primary biliary cirrhosis (PBC), Shakel et al. [54] reported on upregulation of TGF-β2 in PBC, thus providing a further link between TGF-β2 and biliary-derived liver diseases.

In a study including 87 ICC patients, TGF-β2 expression was linked to bad survival and its overexpression in the ICC stroma was significantly associated with ICC classification, microvascular invasion, and the presence of hilar lymph nodes [33], indicating TGF-β2 as a possible prognostic marker for the clinical outcome of ICC and HCC. Recently, a brief report by Fan et al. [55] elegantly used fate tracing experiments to show that cholangiocarcinoma not only originates from biliary cells, but might also develop from a hepatocellular source. These data and our findings demonstrating TGF-β2 expression in diseased liver cells, total liver and HCC lysates and its possibly special role in biliary diseases suggest studying the role of TGF-β2 in hepatocyte-derived cholangiocarcinomas in the future.

In the past, different HCC cell lines where thoroughly investigated regarding the expression of TGF-β1 [39–56]. We were now able to show that almost all cell lines with high TGF-β1 mRNA expression also express high levels of the TGF-β2 isoform (*r* = 0.811; *p* = 0.015, data not shown). Most remarkably, two cell lines (Hep3B and HuH7) secreted even more TGF-β2 than TGF-β1, suggesting that tumorigenic signaling alteration in these cells is significantly affected by TGF-β2 signaling. Tschaharganeh et al. for example report that TGF-β2 expression is specifically downregulated in YAP-deficient HuH7 cells [51].

In other cancer entities, further TGF-β2 dependent tumorigenic mechanisms have been discovered that might also play a role in liver cancer. Thus, TGF-β2 was able to drive tumor cell dormancy in a head and neck squamous cell carcinoma model in cooperation with TGFβR-I, TGFβR-III and p38α/β. Accordingly, in lymph node metastatic cells, pp38, pSmad2 and pSmad1/5 were activated, while CDK4 was reduced by TGF-β2. This was in contrast to the effects of TGF-β1, arguing for independent actions of the two isoforms [57].

This is in line with our Oncomine^®^-based HCC cohort analysis, showing TGF-β2 upregulation in HCC as compared to normal liver, unlike TGF-β1, which was either not regulated or downregulated. Furthermore, this assumption is also valid for our *in vivo* mouse models of HCC. While TGF-β1 was significantly altered in tumors compared to surrounding tissue in DEN-induced HCC, TGF-β2 expression was significantly affected in tumors of TGFα/cMyc mice and DEN-treated mice compared to healthy liver. These data imply etiology specific-regulation of TGF-β2 in liver disease and the importance of the tumor environment. Differential expression of signaling components in normal liver vs. tumor surrounding tissue and vs. tumor tissue has to be analyzed carefully, as tumor-surrounding tissue in the liver can hardly be considered to be healthy and is known to impact tumorigenesis on its own. Consequently, expression changes in the tumor as well as in the surrounding tissue do impact patient survival, while the ratio of the two expression levels does not necessarily reveal survival-relevant information [58].

One further step of complexity is provided to TGF-β isoform specific disease mechanisms by results showing an interdependence of the signaling of the different TGF-β isoforms. In the human cancer cell lines, DU145 (human prostate adenocarcinoma) and A375 (human skin melanoma), Oh and colleagues [59] demonstrated that suppression of TGF-β1 induced TGF-β3 upregulation and therefore they may compensate each other for some, thus far unknown functions. Suppression of TGF-β2 induced downregulation of both, TGF-β1 and TGF-β3 and raises the assumption of a common transcription factor regulated by TGF-β2. Studies by Hellerbrand underlined the fact that the TGF-β2 and -β3 isoforms do not compensate the lack of TGF-β1 in the context of HSC activation *in vivo*, although HSCs were activated, suggesting participation of the other TGF-β isoforms [60]. Not only specific isoform depletion affected TGF-β isoform expression, also TGF-β1 treatment could induce the expression of TGF-β2 via Rho kinase signaling as shown in data from Shimada et al. [36].

In summary, we directly compared the expression dynamics of TGF-β1 and TGF-β2 in the progression of liver disease of different etiologies for the first time in cell lines, animal models, and human patients. Our data suggest that distinct and independent as well as parallel roles exist for the two TGF-β isoforms in liver regeneration and the development of CLD. The significance and the mechanistic details of TGF-β2 regulation and effects are possibly etiology-dependent and have to be delineated. Specific multivariate analyses need to be performed and evaluated in order to make TGF-β2 a promising new candidate and target for the development of liver therapeutics.

## MATERIALS AND METHODS

### Ethics statement

This investigation has been conducted in accordance with ethical standards, the Declaration of Helsinki, and according to national and international guidelines, and has been approved by the authors' institutional review board.

### Cell culture

Human hepatocellular cancer cell lines -HepG2, Hep3B, PLC/PRF, HLE, HLF, FLC-4, and HuH7-, and the human HSC cell line Lieming Xu (LX)-2 [40] were cultured in Dulbecco's modified Eagle's medium (Lonza, Basel) with 10% fetal bovine serum for HCC cell lines, respectively 2% for LX-2 cells, 1% penicillin (100 IU/ml)/streptomycin (100 μg/ml), and 2 mM glutamine. Human hepatoblastoma cell line HuH6 cells were cultured in RPMI 1640 medium (Lonza, Basel) with 10% fetal bovine serum, 1% P/S and 1% glutamine. The cells were maintained in a 37°C humidified atmosphere containing 5% CO_2_.

24 hours prior to the HCC cell line and the HSC line experiments, medium was changed to ‘starvation medium’ with 0.5% FBS. 10 ng/ml TGF-β (PeproTech GmbH, Hamburg, Germany) were used for treatment.

FLC-4 is a derivative cell line of JHH-4, and was obtained by starvation mutation from JHH-4 [61]. Detailed cell line information about JHH-4 can be obtained at http://cellbank.nibio.go.jp/∼cellbank/en/search_res_det.cgi?ID=1365.

### Isolation and culturing of primary murine hepatocytes

Primary hepatocytes were isolated from livers of male C57BL/6 wild type mice by collagenase perfusion [62]. The cells were plated on collagen-coated plates and cultured in Williams E medium supplemented with with 10% fetal bovine serum, 1% penicillin (100 IU/ml)/streptomycin (100 μg/ml), 2 mM glutamine and 0,1% Dexamethasone. After 4 hours of attachment, the cells were overlayed with Williams medium for monolayer or with collagen and medium for sandwich culture. Collagen sandwich culture was performed as previously described [37].

### Isolation of primary mouse HSCs

Primary HSCs were isolated from female BALB/c wild type mice by pronase/collagenase digestion followed by density gradient centrifugation and were cultured on plastic dishes in DMEM, supplemented with 4 mM L-glutamine, 10% FBS, and penicillin (100 IU/ml)/streptomycin (100 μg/ml) as described in [63].

### Reagents

All general chemicals were purchased from Carl Roth GmbH, Karlsruhe or from Sigma-Aldrich Co., USA, unless otherwise stated, and were of the highest quality.

### Animal models

#### CCl_4_-induced liver injury

Acute liver injury was induced in 8-weeks old C57BL/6 mice (4 animals per group) through intraperitoneal injection of 1 ml/kg body weight CCl_4_ (mixed 1:8 with mineral oil), and the mice were sacrificed at 0 h, 3 h, 6 h, day 1, day 2, day 3, or day 6 post injection [64]. For chronic liver injury, C57BL/6 or BALB/c mice received i.p. injections of CCl_4_ (0.7 ml/kg body weight in mineral oil) twice a week for six weeks (*n*_ut_ = 12; *n*_24 h_ = 12; *n*_3x_ = 13; *n*_6wk_ = 12 animals per group). The animals were sacrificed at indicated time points and the liver samples were immediately snap-frozen in liquid nitrogen. Specimen sampling was performed for RNA, protein, serum, and immunohistochemical analyses.

### Bile duct ligation (BDL)–induced cholestatic liver injury

The surgical procedure was carried out as previously described by Abshagen et al. [65, 66]. Under isoflurane anesthesia (1.5 vol%) male BALB/c mice (*n* = 3–5) were placed on a heat pad and laparatomized. The common bile duct was isolated, ligated three times with non-resorbable sutures (polyester 5–0; Catgut, Markneukirchen, Germany), and cut between the two gut-near ligatures. Sham-operated mice underwent a laparotomy with exposure but not ligation of the bile duct (0 h). The abdominal muscle and skin layers were stitched and the mice were treated with metamizole as an analgesic in their drinking water. The animals were allowed to recover from anesthesia and surgery under a red warming lamp and were held in single cages. After 0, 6, 12, 18, or 30 hours or 2, 5, or 14 days mice were killed to obtain blood and liver samples (*n* =3–5).

### MDR2-KO model

*Mdr2*^−*/*−^ mice (MDR2-KO) and control wild type animals were maintained in a specific pathogen-free environment and the experiments were performed with age-matched male mice. Genotyping was done as described elsewhere [67], and liver and blood samples were obtained at the corresponding time points as described previously [68].

### DEN model - chemical-induced liver carcinogenesis

Male mice at 15 days of age received intraperitoneal injections of DEN (5 mg/kg) diluted in saline buffer; the control animals were injected with saline buffer intraperitoneally. At the indicated time points, the mice were sacrificed and their livers removed. Total RNA was isolated from frozen tissues to analyze gene expression by real-time quantitative PCR. Three to four animals per condition and two different tissue pieces per animal were processed for RNA extraction [69].

### TGFα/cMyc transgenic mouse model for hepatocarcinogenesis

Double transgenic TGFα/cMyc mice were generated by crossing c-Myc mice with TGF-α mice as described in [70]. In male TGFα/cMyc mice, hepatocarcinogenesis was triggered by the addition of ZnCl_2_ to the drinking water. Tumor development in the liver was detected by Gd-EOB-DTPA-enhanced magnetic resonance imaging [71]. Tumor and normal liver tissue were dissected and frozen at −80°C until use.

### RNA isolation and cDNA synthesis

Total RNA was extracted according to the manufacturer's instructions using either peqGOLD RNAPure (Peqlab Biotechnologie, Erlangen, Germany) or with Trizol reagent (Life Technologies) and were purified via chloroform extraction or using the High Pure RNA Isolation Kit (Roche). The RNA concentration was determined with the help of the Tecan infinite M200 Microplate reader (Tecan, Switzerland). Subsequently, cDNA was synthesized from 0.5–1 μg RNA with the Transcriptor First Strand cDNA Synthesis Kit (Roche).

### Quantitative real-time PCR

Real-time polymerase chain reaction (qPCR) was carried out with Power SYBR Green (Life Technologies) or TaqMan Universal Master Mix II (Life Technologies) using the Stratagene MX 3005 P system. The primers and probes are listed in supplementary Tables 1 and 2, respectively. To ensure that the primers produced specific PCR amplification products, a dissociation curve was analyzed to guarantee specificity. (Only primers with a unique dissociation peak were selected). To compensate for the variation between qPCR runs, the target gene expression was normalized to the expression of the endogenous, unregulated reference gene rS18 or PPIA. The relative quantity of target genes was determined according to the ΔΔCt (“delta-delta”) method [72].

### High-throughput gene expression analysis using microfluidic fluidigm's biomark HD quantitative chip platform

For high-throughput quantitative Taqman qPCR analysis, we used the microfluidic Fluidigm's BioMark HD high-throughput quantitative chip platform (Fluidigm Corporation, San Francisco, CA, USA) with pre-designed gene expression assays from Life Technologies according to the manufacturer's instructions [73] and as previously described [74]. The data were analyzed using the ΔΔCt method [75] and the expression values were normalized to the expression levels of the housekeeping genes (β-actin, tubulin, GAPDH).

### TGF-β2 inhibition using antisense oligonucleotides (AONs)

HuH7 cells were cultured at medium density to perform AON experiments. TGF-β2 inhibition oligonucleotides were provided by Isarna Therapeutics. AON or scrambled oligonucleotide transfection was performed using RNAiMAX (Invitrogen, Darmstadt, Germany) according to the manufacturer's protocol. Knockdown efficiency was verified by qPCR. The final AON and scrambled control concentration was 20 nmol. Knockdown was allowed to establish for 48 h in medium supplemented with 2% FBS.

### ELISA

Enzyme-linked immunosorbent assays (ELISAs) were performed to estimate the levels of secreted TGF-β1 and TGF-β2 in cell supernatants after normal cell culture. For lysates, the cell culture medium was removed by centrifugation, Cell Lysis Buffer 1 (R … D Systems) was added to the cell pellet and the mixture was allowed to incubate for 60 minutes with gentle agitation. After activating TGF-β through acidification in the collected supernatants or lysates, ELISAs were performed according to the manufacturer's instructions for Quantikine human TGF-β1 or murine TGF-β1 (R … D Systems, Minneapolis, USA) or Quantikine human TGF-β2 or murine TGF-β2 (R … D Systems, Minneapolis, USA), respectively. For measuring TGF-β1 and TGF-β2 secretion of murine HSCs, an ELISA-based system, Luminex, was purchased from BioRad.

### Immunoblot

Proteins were separated by SDS-PAGE, and then electrically transferred onto a nitrocellulose membrane. Primary antibodies against pSmad2, Smad1, Smad2/3 (Cell Signaling Technology), pSmad1/3 (Abcam), and GAPDH (Santa Cruz) were used according to the manufacturer's recommendations. HRP-conjugated secondary antibodies were goat anti-rabbit IgG-HRP or goat anti-mouse IgG-HRP (Santa Cruz). Protein detection was performed using the Super Signal West Dura Extended Duration Substrate (Thermo Scientific). All experiments were performed multiple times.

### Statistical analysis

Error bars represent standard error to the mean. Deviations were used, unless described otherwise; two-tailed Student-t tests or one-way ANOVA were used to calculate the *p*-values. Additionally, Pearson correlation was performed. Differences were considered to be significant if the calculated *p*-value was *p < 0.05, **p < 0.01, ***p < 0.001; the *p*-values were not significant if not indicated.

### Patient collectives

Publicly available databases (iCOD omics.tmd.ac.jp/icod_pub_eng/; Oncomine https://www.oncomine.org/; GEO, http://www.ncbi.nlm.nih.gov/gds; and Arrayexpress, http://www.ebi.ac.uk/arrayexpress/), as well as data generated within the former SFB/TRR77 on Liver Cancer (GSE50579; GEO ID) and those collected from our own collaborative projects, were used in order to analyze expression of TGF-β1 and TGF-β2. The tools Bioconductor (R) 2.13 (3.0.1) and GraphPad Prism 6 were used to retrieve and analyze data from six HCC sample cohorts (GSE5975, GSE1898, GSE50579, iCOD, GSE4024/GSE1898, GSE14520) comprising a total number of 896 HCC patients, presented from different etiologies. The expression values were matched to normal liver samples and/or to each patient's surrounding non-cancer tissue. The following criteria were used at different stages of the screen: (1) significance below 0.05 (*p*-value < 0.05), (2) number of cohorts with significant differences in the TGF-β1 and TGF-β2 expression (≥ 3), and (3) tendency of TGF-β1 and TGF-β2 expression within different cohorts (up- or down-regulated). The Oncomine^®^ Research Edition was used to analyze 7 additional HCC collectives and 7 cirrhosis/precancerous stage collectives for TGF-β1 and TGF-β2 expression (for more details, see Tables 1 and 2). As criteria for analysis of expression changes in HCC vs normal, in cirrhosis/precancerous stages vs normal, and in HCC vs cirrhosis/precancerous stages *p*-values of < 0.05 were set.

## SUPPLEMENTARY MATERIALS FIGURES AND TABLES



## Abbreviations

TGF-β: Transforming growth factor
TGF-βR-I, II, III: Transforming growth factor receptor type I, II and III
CM: collagen monolayer
CLD: chronic liver disease
Col1a1: Collagen 1a1
CS: collagen sandwich
ELISA: enzyme–linked immunosorbent assay
HCC: hepatocellular carcinoma
HSC: hepatic stellate cell
CCL_4_: carbontetrachloride
BDL: bile duct ligation
DEN: diethylnitrosamine
ICC: intrahepatic cholangiocarcinoma
DMEM: Dulbecco's Modified Eagle Medium
KO: knock out
MDR2: multi drug resistance protein 2, phosphatidyl flippase
qPCR: quantitative realtime polymerase chain reaction
SYBR: N′,N′-dimethyl-N-[4-[(E)-(3-methyl-1,3-benzothiazol-2-ylidene)methyl]-1-phenylquinolin-1-ium-2-yl]-N-propylpropane-1,3-diamine
LX-2: human hepatic stellate cell line Lieming Xu-2, HepG2, Hep3B, PLC/PRF, HLE, HLF, FLC-4
HuH7: human hepatocellular carcinoma cell lines
HuH6: human hepatoblastoma cell line
C57BL/6, BALB/c: wild type mouse strains
