# Supplementary material for: TGF-β1 and TGF-β2 abundance in liver diseases of mice and men
Source: Oncotarget. 2016 Jan 21;7(15):19499–518. doi: 10.18632/oncotarget.6967 (PMC4991397; doi:10.18632/oncotarget.6967)
Supplement: Supplementary file 1 [file oncotarget-07-19499-s001.pdf]

TGF-β1 and TGF-β2 abundance in liver diseases of mice and men

Supplementary Materials

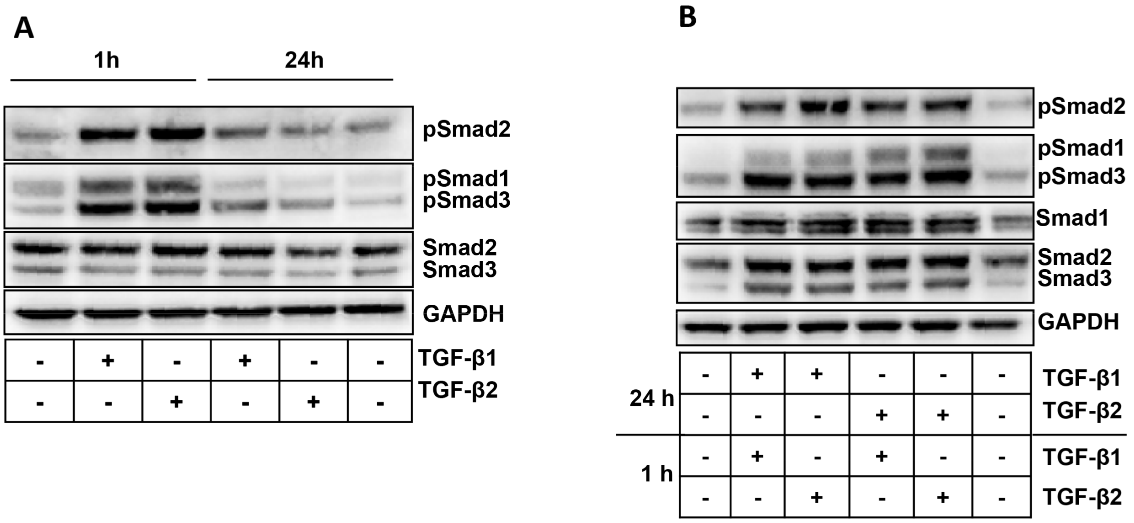

Supplementary Figure S1: Immunoblot analysis of Smad1, 2 and 3 expression and phosphorylation upon stimulation with 10 ng/ml TGF-β1 or TGF-β2 recombinant protein. (**A**) Stimulation time and cytokines were used as indicated in the Figure. In (**B**) the first treatment durated 24 hours, followed by a second stimulation for 1 h.

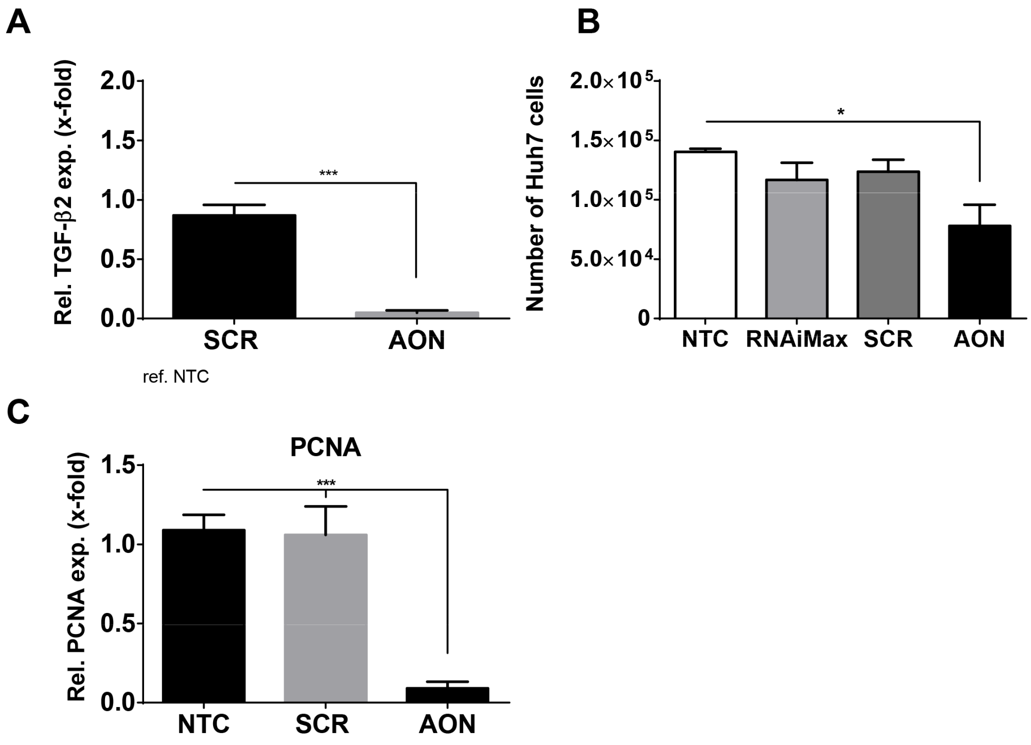

Supplementary Figure S2: AON-mediated knockdown of TGF-β2 in HuH7 cells. (**A**) Knockdown efficiency was confirmed by qPCR after 48h and referred to non-treated cells. (**B**) Impact on proliferation was examined by cell counting and (**C**) PCNA mRNA expression analysis using qPCR.

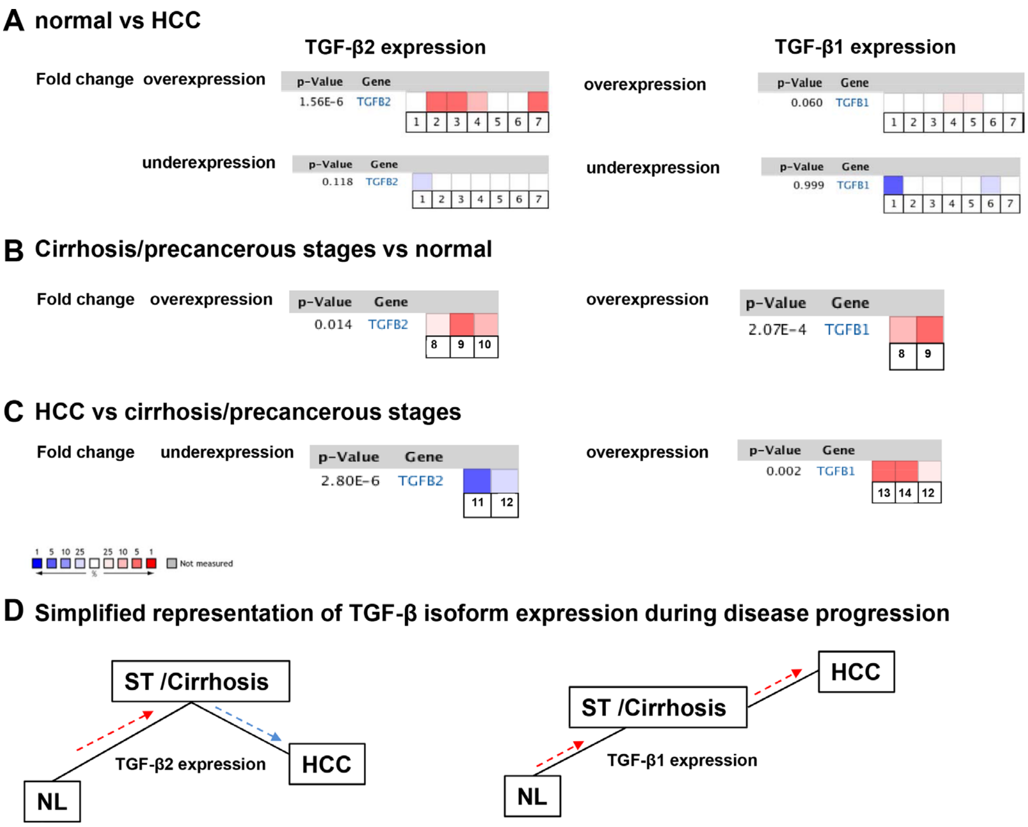

Supplementary Figure S3: TGF-β1 and TGF-β2 expression in human HCC, cirrhosis and precancerous stages using Oncomine® Research database. TGF-β1 (right panels) and TGF-β2 (left panels) expression in human HCC and precancerous stages were examined using *in silico* analysis on the Oncomine® database platform. *p*-values of < 0.05 were set and the p-value for a gene given is its p-value for the median ranked analysis. (**A**) Numbers equal the list of references in Table 1. Gene expression in HCC vs normal liver was analyzed. (**B, C**) Numbers equal the list of references in Table 2. Gene expression in precancerous stages/cirrhosis vs normal or cancer vs precancerous stages/cirrhosis was analyzed. Of note: In the Mas Liver data TGF-β2 was also downregulated significantly ($p = 2.54E-5$, top 23%, median rank 2790) in cirrhosis vs. normal tissue. (**D**) Scheme for potential TGF-β1 and TGF-β2 regulation during disease progression.

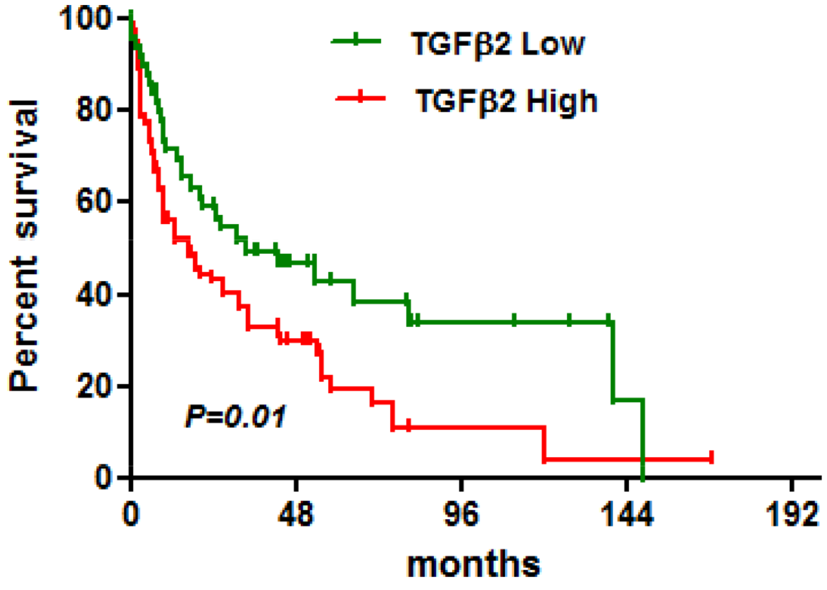

Supplementary Figure S4: Correlation analysis of TGF-β2 expression with survival of HCC patients. 121 patients with defined TGF-β2 values in GSE1898/4024 were subjected to the Kaplan-Meier-plot analysis. High and low expression was assigned according to the median expression levels of all patients.

Supplementary Table 1: qPCR primer based on SybrGreen approach

Gene name	Forward	Reverse	species
rS18	AAACGGCTACCACATCCAAG	CCTCCAATGGATCCTCGTTA	human
PPIA	GAGCTGTTTGCAGACAAAGTC	CCCTGGCACATGAATCCTGG	mouse
TGF-β1	TGGTGGAAACCCACAACGAA	GAGCAACACGGGTTCAGGA	human
TGF-β1	AGGGCTACCATGCCAACTTC	CCACGTAGTAGACGATGGC	mouse
TGF-β2	GCAGATCCTGAGCAAGCTG	GTAGGGTCTGTAGAAAGTGG	human
TGF-β2	GCAGATCCTGAGCAAGCTG	GTAGGGTCTGTAGAAAGTGG	mouse
Col1a1	ACGTGGAAACCCGAGGTATG	TTGGGTCCCTCGACTCCTAC	mouse
PCNA	CTGAGGGCTTCGACACCTAC	TCACTCCGTCTTTTGCACAG	human

Supplementary Table 2: qPCR primer based on Taqman assays. All assays were purchased from Life Technologies® and used according to manufacturer's recommendations

Gene name	Assay ID	species	qPCR type
Endoglin	Hs00923996_m1	human	Taqman
TGF-βRI	Hs00610320_m1	human	Taqman
TGF-βRII	Hs00947893_m1	human	Taqman
TGF-βRIII	Hs01114253_m1	human	Taqman
rS18	Hs03003631_g1	human	Taqman
TGF-β2	Mm00436955_m1	mouse	Taqman
TGF-β1	Mm01178820_m1	mouse	Taqman
